# What’s the way out? Potential exit strategies from the COVID-19 lockdown

**DOI:** 10.7189/jogh.10.010370

**Published:** 2020-06

**Authors:** Aziz Sheikh, Asiyah Sheikh, Zakariya Sheikh, Sangeeta Dhami, Devi Sridhar

**Affiliations:** 1 Centre for Medical Informatics, Usher Institute, The University of Edinburgh, Edinburgh, UK; 2 Medical School, The University of Edinburgh, Edinburgh, UK; 3 General Practitioner Locum, Edinburgh, UK; 4 Centre for Population Health Sciences, Usher Institute, The University of Edinburgh, Edinburgh, UK

With over 4.5 billion people under some form of lockdown, concern is mounting about the economic, social and adverse health effects resulting from these restrictions, including disruption in non-COVID-19 related health care and an increasing number of families being thrown into poverty [1,2]. Policymakers need to balance the clear benefits of lockdown measures in containing transmission of SARS-CoV-2 against growing concerns about their negative effects.

The current UK lockdown measures began on 23 March 2020, following which emergency legislation was passed by the UK Government and its devolved administrations [3]. These regulations are subject to regular review and were extended on 16 April 2020 for a further three weeks [4]. On 17 April 2020, the UK Government outlined five criteria that would need to be met before lockdown measures could be lifted (see **Box 1**). The first two of these criteria have arguably been met as evidenced by the new Nightingale Hospitals remaining largely empty and a decline in the number of daily reported COVID-19 deaths. The remaining three criteria are vaguely articulated, but regardless these have not yet been met. A key omission when compared to the criteria proposed by the World Health Organization (WHO) is the failure to make any mention of active surveillance, tracing and isolation capacity [5].

Box 1 **UK Government criteria for lifting lockdown measures [4].**

1. The NHS must be able to cope.

2. Sustained and consistent falls in daily death rates.

3. Reliable evidence of infection rates decreasing to manageable levels.

4. Confidence that there is sufficient testing capability and personal protective equipment (PPE).

5. Confidence that lifting restrictions will not result in a second peak.

Of prime importance to governments and the public is how and when lockdown can be lifted. Attempts to infer from previous pandemics (eg, H1N1, SARS) are fraught with challenges given continuing uncertainties about parameters as fundamental as the rate of transmission (R or more accurately R_t_, the effective reproduction number) and case fatality rate [6]. Both figures are very difficult to estimate reliably given the lack of a true denominator of the number of cases in the UK [7-9].

To bring clarity for future decisions, we have identified and mapped the range of approaches that are either actively being explored or have been implemented internationally (**Table 1**). The objectives of these approaches are either to: i) promote natural herd immunity; ii) release lockdown measures once a treatment or vaccine become available; iii) attempt to completely eliminate the virus; or iv) find ways of relaxing lockdown whilst still containing virus transmission through a ‘test, trace, isolate’ strategy. Such measures would need to continue until an effective treatment is available or herd immunity (whether naturally or through mass vaccination) develops. We briefly consider each of these approaches in turn.

**Table 1:** Potential COVID-19 lockdown exit strategies

Strategy	Description	Pros and cons	Countries	Supporting evidence
Natural herd immunity	Depending on the true R_0_, this would require between approximately 58%-82% of the population to recover from infection with sufficient levels of antibodies to achieve herd immunity.	Pros: Maintains societal functioning.	Sweden	[10]
		Cons: Reliant on the assumption that long-term immunity is possible despite some evidence of possible reinfections. Large number of deaths in elderly and the vulnerable (akin to ‘survival of the fittest’). Need to stay within health services capacity. Social unrest if individuals unable to access medical services.		
Lockdown till cure or vaccination herd immunity	This option maintains the lockdown indefinitely until a therapeutic is found or a vaccine has been developed, tested, mass-produced and deployed at sufficient levels to achieve herd immunity.	Pros: If maintained, highly likely to prevent transmission.	Current default for many countries	
		Cons: Highly unlikely to be maintainable given social, health & economic consequences. Furthermore, no guarantee that a therapeutic or vaccine will be found.		
Attempt to eliminate the virus	The “test, trace, isolate” strategy advocated by WHO, which aims to fully contain and suppress the epidemic and then reopen society.	Pros: WHO advocated approach with some evidence of success. Keep society and economy running by just removing those carrying virus and breaking chains of transmission.	China, Czech Republic, Faroe Islands, Germany, Iceland, Singapore, South Korea, Vietnam, Greece, New Zealand, Australia	[11]
		Cons: Insufficient surveillance, testing, tracing and isolating capability in the UK. Need for border control to catch imported cases.		[12]
**Containment measures (many of which are likely to be used in combination):**				
Gradual release	Gradual release of lockdown restrictions informed by the areas which are responsible for most economic productivity whilst still being able to adhere to physical distancing measures (eg, construction workers). Needs close monitoring of the number of new cases generated as a result of the release, which would inform whether further loosening or tightening of restrictions is required.	Pros: Allows an assessment of what impact lockdown relaxation measures are having.	Austria, Czech Republic, Denmark, Germany, Italy, Ireland, Norway, Spain	[13]
				[14]
		Cons: May result in actual or perceived inequity leading to social unrest.		[15]
Population scheduling	Splits the population into groups and only allows these groups out at certain days/times. This can allow for the resumption of economic activity whilst helping to maintain physical distancing.	Pros: Allows an assessment of what impact lockdown relaxation measures are having.	Panama; being considered in Croatia, Peru and Spain	[16]
		Cons: May result in actual or perceived inequity leading to social unrest.		
Geographical segmentation	Involves dividing the country into distinct geographical regions and implementing strategies at these regional levels. Could be used selectively to isolate hot spots to prevent widespread transmission through strict enforcement of lockdown measures. Conversely, areas with low transmission would be free to travel within their geographical boundaries.	Pros: Allows an assessment of what impact lockdown relaxation measures are having.	Argentina, Australia, China, Germany, Israel, Italy, Kuwait, Russia, Saudi Arabia, UAE	[17]
		Cons: May result in actual or perceived inequity leading to social unrest. Could lead to movement within countries towards regions with less restrictive policies.		[18]
PPE for the general public	Strongly encouraging or mandating the wearing of PPE in public spaces such as face masks.	Pros: May offer some protection against transmission in areas where physical distancing cannot be maintained such as public transport, shops and workplaces.	China, Singapore South Korea, Scotland, UAE US	[19]
		Cons: Global PPE shortage so may divert essential supplies from the front-line. Face masks should be home-made and cloth-based		[20]

ICU-Intensive care unit, PPE-Personal protective equipment, R_0_ – basic reproduction number, R_t_ – effective reproduction number, UAE-United Arab Emirates, USA – Unites States of America, WHO – World Health Organization

**Figure:**
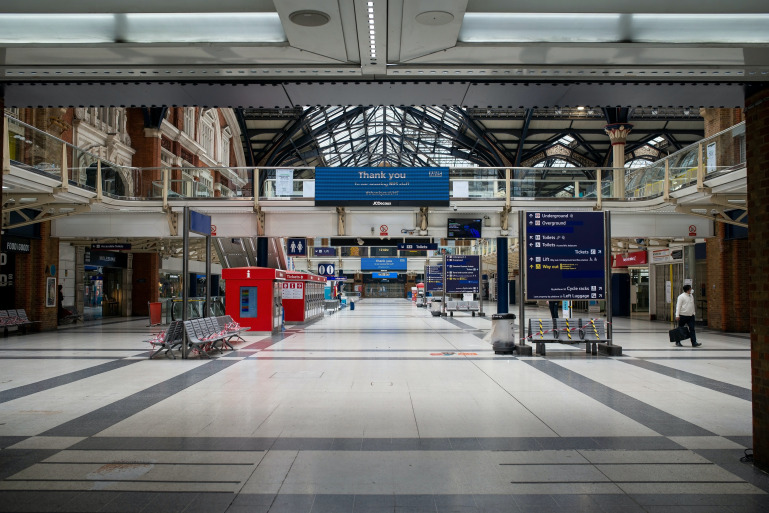
Photo: Liverpool Street Station, London. By Ben Garratt via Unsplash.

The UK, Dutch and Swedish governments initially pursued the idea of promoting herd immunity, but the UK and Dutch have subsequently abandoned this strategy in light of mounting public concerns about the need to infect 60%-80% of the population and revised modelling, which indicated that health systems would be over-whelmed and this could not be pursued as a feasible public health policy [21]. Sweden is in contrast still broadly pursuing this approach with only minimal enforced restrictions on physical movements. Its case is however somewhat atypical as a large proportion of its population ( ~ 40%) live in single person households, it has a highly skilled workforce which enables many people to work from home, and the high levels of education have led to general compliance with messaging about maintaining physical distancing leading to relatively low levels of infection (estimated at ~ 7%) [22]. It is as yet an unproven assumption that long-term immunity is possible despite some evidence of potential reinfections and short-lasting immunity from other coronaviruses [23]. Of note, Sweden’s approach has resulted in significantly more deaths than comparable Nordic countries [24].

Considering the second option, optimistic estimates suggest that a vaccine will not be available for at least 12-18 months and even then it is unknown how safe, effective, available or accessible such a vaccine would be [25]. Given the decimating health, economic and social impacts of lockdowns, it is practically inconceivable that widespread lockdowns can continue even beyond the next 4-6 months. That said, this cannot be a license to recklessly lift lockdown restrictions too early as, for example, the Mayor of Las Vegas was considering [26].

A ‘test, trace and isolate’ strategy is the cornerstone of managing epidemics, and has been repeatedly emphasized by the WHO. Countries such as China (in particular Hubei Province), Hong Kong, South Korea, Taiwan and Vietnam have pursued such approaches achieving substantial control of the disease. Importantly many of these countries had previous experiences of dealing with SARS and MERS, which enabled them to understand the magnitude of the potential challenge very early on and mount effective nationally coordinated multi-sectoral responses. In contrast, the UK and many other Western nations were delayed in instituting their responses, which allowed the numbers of cases to rise very rapidly. Also of relevance in this respect is that these Asian countries there has been active surveillance and enforced isolation of suspected and confirmed cases. Such approaches are far less socially acceptable in Western European contexts. The success of such approaches is also highly dependent on the scale of testing and contact tracing achievable and the efficiency with which test results are turned around. There are therefore a number of substantial challenges that need to be overcome to effectively implement ‘test, trace and isolate’ strategy in the context of the UK [27].

In the UK, the window of opportunity has passed for quick elimination of the virus, given active community transmission as well as the absence of surveillance, testing and tracing capacity. If sufficient political will is found and public health infrastructure is created, it could become a route in the medium- to longer-term. This would however require public acceptance of some of the privacy infringements that contact tracing and an active quarantining approach rely on, which have been employed in a number of other countries. For example, it is estimated that 10 000 new symptomatic cases per day would require 140 000-390 000 contacts to be quarantined/d [28]. Given these requirements, it is currently not feasible to pursue elimination as this would involve maintenance of tight lockdown, vast armies of contact tracers as well as a huge increase in test kits to allow for population testing and extensive quarantining capacity or monitoring of cases.

The last option is we believe the most credible for the UK in the short-term. In essence, this involves finding ways of living with the virus by gradually relaxing some of the lockdown measures whilst simultaneously pursuing aggressive development of active surveillance, testing, tracing and quarantining capacity. These are interim interventions to contain the spread of the disease, keep daily new cases low and minimise its impact until a vaccine, effective treatment, or more data on immunity becomes available. This is likely to involve the careful, gradual opening of segments of society – for example, beginning with predominantly outdoor workers who can maintain physical distancing and/or geographical areas of the country where there is the prospect of eliminating the disease (eg, Scotland’s island communities). This could be followed by a careful phased re-opening of schools with reduced class sizes, desks at 2m distances, face masks for older pupils, frequent hand washing; and the gradual re-opening of non-essential businesses whilst maintaining physical distancing measures. We envisage certain sectors such as bars and restaurants will not open until much later. British Columbia is, for example, considering easing lockdown measures guided by modelling that predicts that increasing social interactions from the current 30% to 40%-60% would not result in a second outbreak [29].

The idea of ‘bubbles’ has been floated whereby each household has interactions with a limited number of others. Recent research has suggested that limited, repeat contacts within the same group may be an effective way of allowing limited socialisation whilst still keeping cases down [30]. This approach does however have the potential to trigger family discord and also has the challenge of being very difficult to monitor and enforce.

Any lockdown relaxation measures need to be considered alongside provision of personal protective equipment (PPE) for those most at risk of acquiring or transmitting the virus and continuing to shield the most vulnerable members of society, including those in care homes. The impact of such changes in policy will need to be continuously monitored using real-time, multi-modal data with a willingness to (temporarily) tighten controls when necessary. Germany recently began to cautiously ease its lockdown measures, a federal rather than national decision, and has very quickly seen a rise in R_t_ from 0.7 to 1, leading to considerations to retighten the restrictions.

What is clear is that there is no straightforward way out and that moving forward will be a delicate balance involving difficult trade-offs. Whichever approach is taken, transparency and clear communication with the public is essential as well as recognition that apprehension might exist to re-engaging with aspects of public life [31].
